# The Thyroid Gland: A Revision Study on Its Vascularization and Surgical Implications

**DOI:** 10.3390/medicina58010137

**Published:** 2022-01-17

**Authors:** Jacopo J. V. Branca, Alfredo Lascialfari Bruschi, Antonino Marcello Pilia, Donatello Carrino, Giulia Guarnieri, Massimo Gulisano, Alessandra Pacini, Ferdinando Paternostro

**Affiliations:** Department of Experimental and Clinical Medicine, Anatomy and Histology Section, University of Firenze, L.go Brambilla 3, 50134 Firenze, Italy; jacopojuniovalerio.branca@unifi.it (J.J.V.B.); a.lascialfaribruschi@virgilio.it (A.L.B.); marcello.pilia@outlook.it (A.M.P.); donatello.carrino@unifi.it (D.C.); giulia.guarnieri@unifi.it (G.G.); massimo.gulisano@unifi.it (M.G.); alessandra.pacini@unifi.it (A.P.)

**Keywords:** thyroid gland, thyroid arteries variations, superior thyroid artery, inferior thyroid artery, thyroid ima artery

## Abstract

*Background*: The “classic” thyroid gland arterial vascularization takes into account two superior thyroid arteries (STA), two inferior thyroid arteries (ITA) and, occasionally, a thyroid ima artery (TIMA). The present review focuses on exploring the available data concerning thyroid gland arterial vascularization and its variations. *Methods*: Here, we analysed 49 articles from the last century, ranging from case reports to reviews concerning cadaver dissection classes, surgical intervention, and non-invasive techniques as well. *Results*: The harvested data clearly highlighted that: (i) the STA originates predominantly from the external carotid artery; (ii) the ITA is a branch of the thyrocervical trunk; and (iii) the TIMA is a very uncommon variant predominantly occurring to compensate for ITA absence. *Conclusion*: A systematic review of a highly vascularized organ is of great relevance during surgical intervention and, thus, the knowledge of normal anatomy and its modification is essential both for fact-finding and in surgery.

## 1. Introduction

The thyroid gland is a richly vascularized endocrine gland [1]. It is an uneven organ located in a median position in the antero-lower region of the neck, between the fifth cervical vertebra and the first thoracic vertebra.

The importance of studying the vascularization of the thyroid gland and its variation is mainly due to its close relationship with other structures, especially muscle and vascular nerve bundles.

Topographically, the medial face of the thyroid gland is placed directly on the ventral surface of the larynx and the trachea, while the ventral surface of the gland, starting from the anterior and medial face of the organ, is covered by the subhyoid muscles (such as the sternothyroid, homohyoid and sternohyoid muscle) that are enclosed by the splitting of the middle cervical fascia.

Laterally, in correspondence of the lobes, there are the sternocleidomastoid muscle and, more superficially, the superficial cervical fascia and the platysma. Furthermore, the lateral surface of the two lobes is related to the vascular nerve bundle of the neck, covered by the carotid sheath holding the common carotid artery, the internal jugular vein, and the vagus nerve. Moreover, medially, between the thyroid gland sheath and the oesophagus–tracheal corner, it is worth noticing the recurrent laryngeal nerve, closest to the left lobe.

Concerning the thyroid isthmus, it is superficially covered by the sternohyoid muscle and, laterally, by the anterior jugular veins.

Finally, between the peri-thyroid sheath and the capsule that adheres to the parenchyma, the so-called “dangerous space” must remain intact when the gland is removed in order to avoid severe bleeding, since it holds the rich thyroid venous network.

For what may concern the blood supply, it is achieved through two superior thyroid arteries (STA), two inferior thyroid arteries (ITA), and sometimes by an additional artery, the thyroid ima artery (TIMA) that reaches the gland from below at the isthmus level.

By-the-book, the STA takes origin as first branch from the external carotid artery (ECA), at the height of a horizontal plane passing through the hyoid bone. Concerning its origin, it is behind the sternocleidomastoid muscle and it is in relationship, medially, with the inferior pharyngeal constrictor muscle, the larynx, and with the superior laryngeal nerve. Furthermore, different branches such as the infrahyoid, sternocleidomastoid, superior laryngeal, and cricothyroid artery originate from the STA.

On the other hand, the ITA originates as terminal artery from the thyrocervical trunk (TCT) that arises, as a short and wide artery, from the front of the first part of the subclavian artery (SA). The relationship between the ITA and the recurrent laryngeal nerve is extremely important since the ITA usually passes behind the nerve. However, closest to the thyroid gland, the right nerve is equally likely to be anterior, posterior or amongst, and the left nerve is usually posterior. Furthermore, on the left side, near its origin, the artery is crossed anteriorly by the thoracic duct as the latter curves infero-laterally to its termination. Furthermore, collateral branches arise from the ITA: the ascending cervical and the inferior laryngeal artery, and the oesophageal, pharyngeal and tracheal branches [2,3].

However, part of the scientific literature tends to deny this “classic” origin and shows numerous anatomical variants.

Indeed, over the years, several studies (on cadaver models or through non-invasive imaging techniques) and case reports have demonstrated the anatomical variations due to the presence of the abovementioned arteries. 

For these reasons, in this review, we collected and described all these variants in more detail as possible.

## 2. Methods

In the present study, 49 articles have been analysed, including clinical case reports (surgical intervention and non-invasive techniques), reviews and cadaver dissection classes concerning thyroid gland vascularization, together with meta-analyses and studies on different populations. 

Concerning the anatomical data, the main parameters analysed were the presence, origin and symmetry of the thyroid arteries together with the distance between the STA origin, and the carotid bifurcation (CB). Furthermore, concerning the STA, the external diameter was also been taken into account.

Concerning the inclusion criteria, no limitations (including temporal range) were set for the research, to analyse a wider range of cases.

The present revision study was conducted searching articles present in the MEDLINE database by the free search engine Pubmed.

## 3. Results

### 3.1. The Origin of the Superior Thyroid Artery (STA)

One of the first variants characterizing the STA is its origin.

In fact, in 24 studies analysed, ranging from 1974 to 2019, the STA showed the origin not only from the ECA, but also at the level of the CB or from the common carotid arteries (CCA). These studies, reported in Table 1, were mainly conducted on cadavers, but the data were also obtained in vivo by non-invasive techniques (angiography) or as case reports during surgical intervention, taking also into account different populations or ethnicities [4,5,6,7,8,9,10,11,12,13,14,15,16,17,18,19,20,21,22,23,24,25,26,27].

The two more conspicuous studies were published by Vázquez and colleagues in 2009 [6] and by Esen and colleagues in 2018 [20], based on 165 cadavers (207 heminecks) and 640 patients by a tomographic method, respectively.

The analysis provided by Vázquez and colleagues reported that in 49% of cases the STA originated from the CB, whereas Esen and colleagues found in most cases the origin was at the ECA level, more precisely in 64.5% of cases (*n* = 413) on the right and in 39.7% of cases (*n* = 254) on the left.

Moreover, 12 of the aforementioned studies also analysed the distance from the CB when the STA originated from the CCA or the ECA. In this regard, the distances from the CB ranged from 1 to 35 millimetres (mm) when the STA arose from the CCA, and ranged from 1 to 18 when the origin was from the ECA, as reported in Table 2 [4,5,6,8,10,12,15,18,21,22,23,24].

Similar cases are those described by Smith and colleagues in 1978 [28]. During the dissection of an African American man, it was revealed that the right STA originated from the CCA at a distance of 27 mm from the bifurcation; Adachi and colleagues found a distance of 25 mm [29], whereas others found a 12 mm distance of STA from CB [30], thus reconsidering the previous Livini statement claiming that the STA rarely originates from more than 5 mm proximal to the CB [31].

Finally, a particular and rare case was reported in 2010 by Mehta and colleagues who, during a dissection, discovered an absence of the right STA [22].

However, it should be mentioned that this deficiency is filled by the contralateral STA, which has its origin at the CCA level, with a distance from the CB of 0.5 cm (5 mm). It is also useful to note that the STA in the left hemineck replaces the lack of the right one by moving towards the upper poles of the thyroid gland as an inverted Y, thus compensating for the vascularization of the upper portion.

### 3.2. STA Common Trunk (Lingual and Facial Arteries)

Still regarding the STA’s possible anatomical forms, and more generally the variants involving the ECA’s branches, there are as many studies that analysed the origin of the STA as a common trunk with other arterial vessels, such as the lingual artery (LA) and the facial artery (FA), reported in Table 3 [5,6,7,8,9,10,13,14,16,18,19,32].

In this regard, it is worth mentioning the study conducted on 330 heminecks by Vázquez and colleagues in 2009 describing one more of STA’s origins, classified as type IV, from a common trunk with LA: namely, a thyrolingual trunk (subgroup IVa) or a thyrolinguofacial trunk (subgroup IVb) with a frequency of 0.6% and 0.3%, respectively [6].

**Figure 1:**
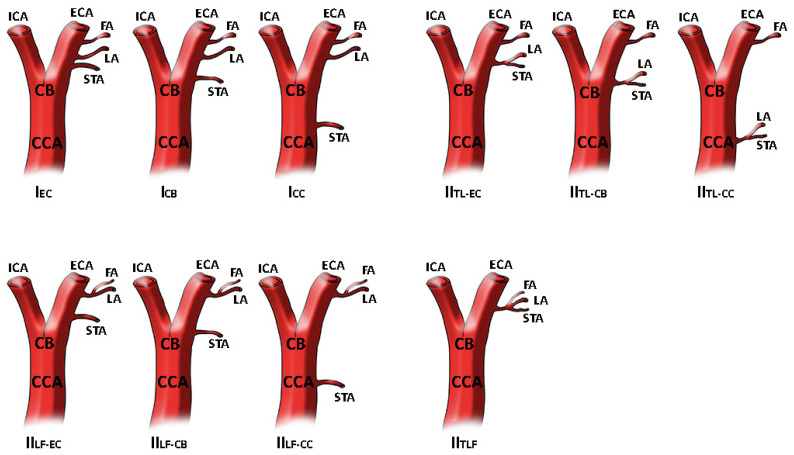
STA different origin as reported and modified by Natsis and colleagues. In type I STA origins as separate branch at different levels. In type II STA is a part of thyrolingual trunk or thyrolinguofacial trunk. The image has been modified as previously reported [7]. Superior thyroid artery origin classification. Type I, separate branches, I_EC_: STA as separate branch from ECA, I_CB_: STA as separate branch from CB, I_CC_: STA as separate branch from CCA. Type II_TL_ branching patterns, II_TL-EC_: thyrolingual trunk from ECA, II_TL-CB_: thyrolingual trunk from CB, _IITL-CC_: thyrolingual trunk from CCA. Type II_LF_ branching patterns, II_LF-EC_: linguofacial trunk and STA from ECA, II_LF-CB_: linguofacial trunk from ECA and STA at the level of CB, II_LF-CC_: linguofacial trunk from ECA and STA from CCA. Type II_TLF_ branching pattern, thyrolinguofacial trunk from ECA.

In addition, the analysis conducted in 2011 by Natsis and colleagues [7], described the origin of the STA as part of a common trunk with the LA with no case found for the thyrolinguofacial trunk. However, Natsis and colleagues proposed a new classification system with two main branching patterns, as reported in Figure 1.

Such an anatomical variation was also reported in previous studies as clearly quoted by Lemaire and colleagues, but only one case, in 1932, was superimposable to their results describing that the thyrolingual trunk arose from the CCA, 30 mm below the CB [33].

Still regarding data concerning the STA, different authors have found differences in the vessel calibre, as reported in Table 4. Indeed, it was found to have an external diameter ranging from 1.04 to 4.4 mm [5,6,15,24,34].

### 3.3. Inferior Thyroid Artery (ITA)

Regarding the ITA, and referring to the existing literature, there are more examples of anatomical variants than the STA, discovered by non-invasive techniques, cadaver dissection or during surgical intervention. The knowledge of the ITA’s anatomical variants is essential for its relationship with the laryngeal nerve: it may be absent, present as an ancillary (accessory) ITA, and appearing double or originate at different levels [20], and as reported in the studies described in Table 5, even if the most common origin of the ITA is the thyrocervical trunk (TCT), it can also originate from other vessels such as the CCA, the subclavian artery (SA), the vertebral artery (VA) or the suprascapular artery (SSA) [20,25,35,36,37,38,39,40,41,42].

Moreover, to be more detailed, one of the first reported cases concerning the ITA’s absence dates back to 1980. The case reported by Krudy [43], performed through a radiographic image of the subject n. 3, shows the ITA’s absence on the left and a hypo-plastic ITA on the right in addition to the TIMA which, plays a fundamental role for the correct gland’s vascularization in this particular case.

The data relating to the study of Yilmaz and colleagues was also remarkable when during a normal neck base dissection, they found an absence of both ITAs.

In this case, the STA does not compensate for these missing vessels, but rather the TIMA does, characterized in this case by larger dimensions than the usual [44].

In this study, the TIMA takes origin from the brachiocephalic trunk, then it protrudes upwards where it splits at the base of the thyroid into right and left branches.

Then the branches enter into the thyroid lobes, thus bringing the necessary vascularization to the gland.

At this point, a particular case report should also be mentioned. Weiglein and colleagues in 1996 described the absence of both ITAs, but they also observed a completely anomalous compensation supply. Indeed, the right ITA was replaced by a TIMA coming from the internal thoracic artery of the same side and, crossing the SA, led to supply the lower thyroid lobe [45].

On the left, however, the ITA was replaced by a branch coming from above, even if its origin was the left vertebral artery (VA) (due to the complete absence of the thyrocervical trunk). This artery was defined by the authors themselves as the supreme thyroid artery.

This particular anatomical variant is the result of an anomaly that occurred during embryonic development. Indeed, the hypothesis takes into consideration a direct connection between the vertebral chain (placed dorsally during the development) and a chain between the cervical artery, the thyrocervical trunk (cranially) and continues downwards (caudally) with the internal thoracic artery.

An ancestral anastomosis between the latter chain of vessels, defined by the authors as cervical longitudinal ventral anastomosis, and the standard vertebral chain, would be the basis of this variant: that is, the TIMA from the internal thoracic artery and the supreme thyroid artery from VA [45].

Another case worth reporting is that of Jelev and colleagues in 2001. They evaluated 102 cadavers and found variations of the branches departing from the SA in one of theme. Specifically, the ITA’s absence was at the right side without being replaced with the TIMA [34].

It follows that the vascular compensation of the organ is provided by the ipsilateral STA, which therefore appears to have a larger calibre (∅ = 4.4 mm) than that previously reported in Table 4. No further variations compared to the classic anatomical schemes were found. On the other hand, it should also be considered that the veins, generally coupled to the arterial vessels, disappear in the right hemineck where the ITA is missing.

### 3.4. Thyroid Ima Artery (TIMA)

Among the first data in the scientific literature evaluating the presence of TIMA, there is the study of Fujimoto and colleagues [24], previously mentioned for the STA. According to their analysis, the authors found a TIMA that supplied the anterior and posterior face of the isthmus with its branches, coming from the brachiocephalic trunk.

From the analysis of 75 Japanese cadavers, it emerges that in 5.7% of the subjects, the TIMA came from the aortic arch, while in 74.3% it came from the brachiocephalic trunk, in 14.3% it originated from the right CCA and, finally, in 5.7% from the right internal thoracic artery.

Another noteworthy work was that of Krudy and colleagues in 1980, previously quoted for the ITA absence. The study analysed 200 subjects, examined through arteriography. Three of them appear to have had the TIMA supply the parathyroid glands [43].

In one case, the TIMA originated from the brachiocephalic trunk. In the second case it originated from the CCA on the right side. On the other hand, the third case presented both a TIMA origin from the brachiocephalic trunk and a right hypoplastic ITA that was missing on the left side. As further confirmation of how TIMA is necessary as “compensation” for a poor classical vascularization (STA/ITA) of the thyroid gland, we have to mention the “isolated phenomenon” found by Moriggl and colleagues [46].

In fact, the authors noted the absence of three thyroid arteries—the two ITAs and the left STA—while the right STA still existed, originating from the CCA moving forward to the thyroid isthmus.

In addition to this discrepancy, the authors also paid attention to the very large diameter of the first portion of the internal thoracic artery and how the same split into two branches once it reached the intercostal space.

**Figure 2:**
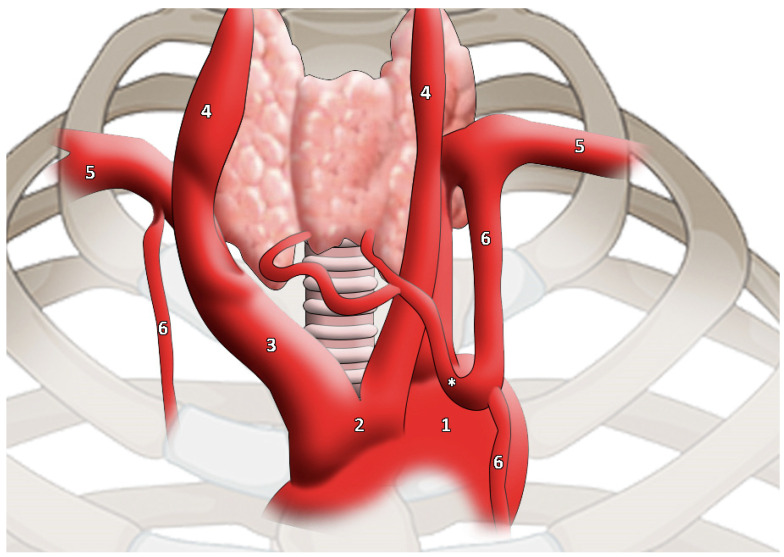
TIMA (highlighted by * asterisk) as reported and modified by Moriggl and colleagues. It is worth noticing the artery calibre [46]. 1, aortic arch; 2, common origin of innominate artery and left common carotid artery; 3, innominate artery; 4, common carotid artery; 5, subclavian artery; 6, internal thoracic artery.

The main branch went to the lower edge of the thyroid gland, subsequently dividing into two branches to supply the gland itself. The artery that compensates for the lack of normal thyroid vessels is called the TIMA, and it should not be considered as an accessory thyroid artery (Figure 2).

The work of Sannomiya and colleagues [47], who found in a cadaver a double TIMA without any particular variation concerning the classic thyroid arteries, was also of particular interest.

In fact, the right and left TIMAs originated from the respective internal thoracic arteries: these, after reaching the interiors of the chest for a few mm, gave rise to the TIMAs that went up and medially, thus supplying the respective right and left thyroid lobes.

This case, along with a few others, testifies a double vascularization of the TIMA; in fact, as the authors themselves cited, only 10 other cases before them had brought to light this double anomalous evidence.

The authors also mention the classification of Niida and colleagues [48], referring to this double variant of the TIMA. In fact, three subgroups are described: type 1 (brachiocephalic trunk + CCA), type 2 (brachiocephalic trunk + aortic arch), and type 3 (right internal thoracic artery + left internal thoracic artery) [47]. Although the presence of a TIMA can be associated with the absence of ITA [43], the scientific literature shows that the two arteries are not necessarily mutually exclusive.

Indeed, Esen and colleagues [20], thanks to a non-invasive study of CT angiography on the carotid arteries conducted on 640 patients, showed that out of 15 individuals (2.3% of cases), TIMA was present, and that for 7 of these cases (46% of cases) it was present together with ITA. In addition, TIMAs originated in most subjects from the brachiocephalic trunk (12/640).

Lovasova and colleagues also confirmed this origin, reporting the presence of TIMA originating from the brachiocephalic trunk, at a distance of 99.8 mm from the CB [25].

Finally, in 2018 Yohannan and colleagues described the TIMA’s origin from the SA close to the VA’s origin, stressing that the TIMA definition was completely arbitrary, in particular when defining its origin [49].

In any case, TIMA has been defined for its characteristic of moving to the trachea front face, originating on the right and supplying the lower lobes and the isthmus of the thyroid gland.

### 3.5. Meta-Analysis on Thyroid Vascularization

Toni and colleagues [50] conducted a meta-analytical study investigating the differences in various ethnic groups (Caucasians and Asians). Results showed the STA’s presence in 99% (*n* = 2079) of the Caucasian subjects (items examined *n* = 2092) examined and in 100% (*n* = 467) of the Asian subjects. Concerning the ITA, it was present in 98% of Caucasian cases (*n* = 2844) and in 93% of Asian subjects (*n* = 532). The reason why STA is more constant lies in the fact that it is the first and “original” vessel of the thyroid rough [50].

As for TIMA, however, it is more present in the Asian ethnicity than in the Caucasian one.

As far as the origin of the STA is concerned, the data collected so far shows that the origin, found mainly on the right side, comes from the ECA, followed by the CCA with a prevalence on the left side. These asymmetries, particularly findable on the right side of the neck, are also found with regard to the ITA, which has a greater possibility of origin from the thyrocervical trunk (90% of cases; *n* = 2127 on a total of 2361 Caucasian subjects), against 95% of cases in subjects Asians (*n* = 260 out of a total of 275), followed by an origin of the SA, the VA and in rare cases by the CCA. The data regarding STA and ITA are confirmed by the studies of the same research group [51,52].

## 4. Discussion

From the data collected so far in the scientific literature regarding the anatomical variants in different districts of the human body [53,54], it emerges how important these evidences are both from a clinical and (specifically) surgical point of view.

Actually, in order to perform successful surgical interventions, a deep knowledge of the anatomical structures is essential and, consequently, the knowledge of possible variants is a must for an excellent surgeon (or health giver).

The lack of knowledge of a junior surgeon in his early years of practice and thus limited experience on the possible variations of origin, calibre, absence or replacement with other vessels in the arterial and venous circulatory system can lead to dramatic bleeding scenarios and sometimes, inevitably, to the patient’s death.

However, in addition to the possible bleeding causes, there is also the dramatic possibility of nerve laceration. This is not just about the renowned recurrent laryngeal nerve, the external branch of the upper laryngeal nerve can also be damaged. In fact, the latter, when detached from the common portion of the superior laryngeal nerve and split into the internal and external branch, then descends together with the STA, which is therefore considered a landmark for identifying the same external branch of the nerve.

The identification of possible thyroid arteries variants, but also other arterial vessels, can be carried out not only through sectorial (dissection) practice but also through non-invasive practices such as angiographic CT (computed tomography) [20], thus allowing both the identification of anatomical variants and also the localization of anomalies.

For example, in possible bleedings that can occur during head–neck carcinoma removal or tonsillectomies, the ligation of the ECA above the STA is frequent.

In some case reports, however, bleeding due to an STA injury cannot be stopped by simply tying the ECA because the STA originates below the CB by 3 cm [14,18].

It follows that a good and correct knowledge of the STA’s possible anatomical variants represents a great help in all surgical practices on the neck, in order to avoid possible iatrogenic damage.

Of certain importance are also the chemoembolization techniques which selectively use carotid artery vessels for chemotherapy administration (with the presence of a head and neck cancer); therefore, a deep knowledge of these vessels and their branches is essential for a successful intervention [5,15].

Vessel calibre is also a critical aspect because it is possible to find an STA with a large diameter and comparable with the upper laryngeal artery’s calibre and this can lead to a ‘confusing’ situation in the operating room, especially in those cases where the upper laryngeal artery originates as the first branch from the ACC [6].

With regard to ITA, in addition to bleeding events, the knowledge of its possible variants is fundamental when concerning the lower laryngeal nerve.

The ITA origin from the CCA could lead to a greater probability of injuries at the nerve level itself [37].

Finally, it is also necessary to pay attention when the ITA is missing: in this case, it is necessary to keep in mind those arteries that can supply the thyroid gland.

For example, it is clear that if the vascular supply is provided by the STA and the latter results obstructed, the arterial supply will cease [39].

No less important are the diagnostic or interventional (or both) procedures (for example a tracheostomy) that can lead to significant bleeding if the presence of a TIMA is not known [46,49].

## 5. Conclusions

Concluding, a correct intervention requires an exact artery localization and a good knowledge of all its possible variants [23], since many mistakes made come from the poor anatomical knowledge of the neck [55].

## Figures and Tables

**Table 1:** Available data concerning STA origin.

Author(s)	Type of Study	Number of Cases	ECA (%)	CB (%)	CCA (%)
(Lučev et al., 2000)	Cadavers	40	30	22.5	47.5
(Ozgur et al., 2008)	Cadavers	40 *	25	40	35
(Vázquez et al., 2009)	Cadavers	207 *	23	49	26.6
(Natsis et al., 2011)	Cadavers	100	39	49	12
(Anagnostopoulou and Mavridis, 2014)	Cadavers	68	23.5	4.4	17.6
(Sanjeev et al., 2010)	Cadavers	37	64.86	-	35.14
(Ozgur et al., 2009)	Cadavers	40 *	25	40	35
(Gupta et al., 2014)	Angiography	25	72	20	8
(Won et al., 2011)	Cadaver (clinical case report)	1	-	-	100
(Ongeti and Ogeng’o, 2012)	Cadavers	46	80.4	2.2	10.9
(Zümre et al., 2005)	Cadavers	40 *	25	70	5
(Troupis et al., 2014)	Cadaver (clinical case report)	1	-	100 (right)	100 (left)
(Nochikattil, 2017)	Surgery (clinical case report)	1	-	-	100
(Motwani and Jhajhria, 2015)	Cadaver (clinical case report)	1	100	-	-
(Lemaire et al., 2001)	Cadaver (clinical case report)	1	-	-	100
(Jadhav et al., 2011)	Cadaver (clinical case report)	1	-	100 right	-
(Esen et al., 2018)	TC angiography	640	64.5 (right) and 39.7 (left)	20.5 (right) and 23.1 (left)	14.1 (right) and 35.3 (left)
(Issing et al., 1994)	Surgery (clinical case report)	1	-	-	100
(Mehta et al., 2010)	Cadaver (clinical case report)	1	-	-	100 (right)
(Dhindsa and Sodhi, 2014)	Cadaver (clinical case report)	1	100 (right)	-	100 (left)
(Fujimoto et al., 1974)	Cadaver (clinical case report)	1	-	-	100
(Lovasova et al., 2017)	Cadaver (clinical case report)	1	100	-	-
(Sreedharan et al., 2018)	Cadavers	60 *	88.33	8.33	3.33
(Hayashi et al., 2019)	Surgery (clinical case report)	1	100	-	-

The results are reported as percentage (%) of cases for different type of study. Asterisk (*) means that the analysis was conducted on heminecks. ECA: origin from the external carotid artery; CB: origin from the carotid bifurcation; CCA: origin from the common carotid artery.

**Table 2:** Available data concerning STA origin and distance from CB.

Author(s)	Type of Study	Number of Cases	CCA-CB (mm)	ECA-CB (mm)
(Vázquez et al., 2009)	Cadavers	207 *	1–21	1–15
(Lučev et al., 2000)	Cadavers	40	2–10.7	2–10.5
(Ozgur et al., 2008)	Cadavers	40 *	-	3.29 ± 4.27
(Anagnostopoulou and Mavridis, 2014)	Cadavers	68	1–6	2–18 (right) and 2–10 (left)
(Ozgur et al., 2009)	Cadavers	40 *	3.29 ± 4.27	3.29 ± 4.27
(Won et al., 2011)	Cadaver (clinical case report)	1	5.3 (right) and 6.4 (left)	-
(Troupis et al., 2014)	Cadaver (clinical case report)	1	10.1	-
(Lemaire et al., 2001)	Cadaver (clinical case report)	1	30	-
(Issing et al., 1994)	Surgery (clinical case report)	1	35	-
(Mehta et al., 2010)	Cadaver (clinical case report)	1	5	-
(Dhindsa and Sodhi, 2014)	Cadaver (clinical case report)	1	14	-
(Fujimoto et al., 1974)	Cadaver (clinical case report)	1	4.5 (right) and 30 (left)	-

The results from different studies are reported in millimetres (mm) as distance from CB distinguish the STA origin both from CCA and ECA. Asterisk (*) means that the analysis was conducted on heminecks. CCA-CB: distance from carotid bifurcation when STA origins from common carotid artery; ECA-CB: distance from carotid bifurcation when STA origins from external carotid artery.

**Table 3:** Available data concerning the STA origin from thyrolingual and thyrolinguofacial trunk.

Author(s)	Type of Study	Number of Cases	Thyrolingualtrunk (%)	Thyrolinguofacialtrunk (%)
(Ozgur et al., 2008)	Cadavers	40 *	2.5	-
(Vázquez et al., 2009)	Cadavers	330 *	0.9	0.3
(Natsis et al., 2011)	Cadavers	100	3	-
(Kapre et al., 2013)	Cadavers	21	9.5	-
(Anagnostopoulou and Mavridis, 2014)	Cadavers	68	14.7	12.3
(Sanjeev et al., 2010)	Cadavers	37	2.7	-
(Ozgur et al., 2009)	Cadavers	40	2.5	-
(Ongeti and Ogeng’o, 2012)	Cadavers	46	6.5	-
(Zümre et al., 2005)	Cadavers	40 *	2.5	2.5
(Nochikattil, 2017)	Surgery (clinical case report)	1	100	-
(Lemaire et al., 2001)	Cadaver (clinical case report)	1	100	-
(Jadhav et al., 2011)	Cadaver (clinical case report)	1	100	-

The results are reported as percentage (%) of cases for different type of study. Asterisk (*) means that the analysis was conducted on heminecks.

**Table 4:** Available data concerning the STA calibre.

Author(s)	Type of Study	Number of Cases	STA Diameter (mm)
(Troupis et al., 2014)	Cadaver (clinical case report)	1	1.04
(Ozgur et al., 2008)	Cadavers	40 *	3.53 ± 1.17
(Vázquez et al., 2009)	Cadavers	207 *	2.6 ± 1.2
(Fujimoto et al., 1974)	Cadaver (clinical case report)	1	3.3 (right) and 2.7 (left)
(Jelev and Surchev, 2001)	Cadavers	1/102	4.4

The results are reported in millimetres (mm) concerning the external diameter of the STA, as analysed in different studies. Asterisk (*) means that the analysis was conducted on heminecks.

**Table 5:** Available data concerning the ITA origin.

Author(s)	Type of Study	Number of Cases	TCT Right	TCT Left	SA Right	SA Left	VA	CCA Right	CCA Left	SSA
(Esen et al., 2018)	TC angiography	640	95	90.3	2.8	2	0.6	-	-	-
(Roshan et al., 2015)	Cadavers	100 *	96	100	4	-		-	-	-
(Hölbling Patscheider et al., 2011)	Cadaver (clinical case report)	1	-	100	100	-	-	-	-	-
(Ngo Nyeki et al., 2016)	Surgery (clinical case report)	1	-	100	-	-	-	100	-	-
(Mariolis-Sapsakos et al., 2014)	Surgery (clinical case report)	1	-	-	-	-	-	100	100	-
(Simmons et al., 1987)	Surgery (clinical case report)	1		-	-	100	-	100	-	-
(Sherman and Colborn, 2003)	Cadaver (clinical case report)	1	100	-	-	-	-	-	-	-
(Cigali et al., 2008)	Cadaver (clinical case report)	1	-	100	-	-	-	-	-	100 (left, accessory)
(Lovasova et al., 2017)	Cadaver (clinical case report)	1	100	-	-	-	-	100	-	-
(González-Castillo et al., 2018)	Cadaver (clinical case report)	1	-	100	-	-	-	-	-	-

The results are reported as percentage (%) of cases for different type of study. Asterisk (*) means that the analysis was conducted on heminecks. TCT: origin from thyrocervical trunk (right or left); SA: origin from subclavian artery (right or left); VA: origin from vertebral artery; CCA: origin from common carotid artery (right or left); SSA; origin from suprascapular artery.
